# Presacral malakoplakia presenting as foot drop: a case report

**DOI:** 10.1186/s13256-023-03883-4

**Published:** 2023-04-07

**Authors:** Tom A. Yates, Katie Devlin, Abed Arnaout, William Hurt, Neil Stone, Kate V. Everett, Alan Pittman, Hardik Patel, Susan Heenan, Paul Hart, Thomas S. Harrison

**Affiliations:** 1 Clinical Infection Unit, Clinical Academic Group in Infection and Immunity, St George’s University Hospitals NHS Foundation Trust, London, UK; 2 Division of Infection and Immunity, Faculty of Medicine, University College London, UCL Cruciform Building, London, WC1E 6BT UK; 3 Radiology Department, St George’s University Hospitals NHS Foundation Trust, London, UK; 4 Department of Neuroradiology, St George’s University Hospitals NHS Foundation Trust, London, UK; 5 Department of Histopathology, St George’s University Hospitals NHS Foundation Trust, London, UK; 6 Hospital for Tropical Diseases, University College Hospital, London, UK; 7 Genetics Research Centre, St George’s University of London, London, UK; 8 Department of Neurology, Epsom and St Helier NHS Trust, St George’s University Hospitals NHS Foundation Trust, London, UK; 9 Institute for Infection and Immunity, St George’s University of London, London, UK

**Keywords:** Malakoplakia, Peripheral nervous system, Infectious diseases, Immunopathology, Human genetics, Ciprofloxacin, Bethanechol, Ascorbic acid, Case report

## Abstract

**Background:**

Malakoplakia is a rare condition characterized by inflammatory masses with specific histological characteristics. These soft tissue masses can mimic tumors and tend to develop in association with chronic or recurrent infections, typically of the urinary tract. A specific defect in innate immunity has been described. In the absence of randomized controlled trials, management is based on an understanding of the biology and on case reports.

**Case presentation:**

Here we describe a case of presacral malakoplakia in a British Indian woman in her late 30s, presenting with complex unilateral foot drop. Four years earlier, she had suffered a protracted episode of intrapelvic sepsis following a caesarean delivery. Resection of her presacral soft tissue mass was not possible. She received empiric antibiotics, a cholinergic agonist, and ascorbic acid. She responded well to medical management both when first treated and following a recurrence of symptoms after completing an initial 8 months of therapy. Whole exome sequencing of the patient and her parents was undertaken but no clear causal variant was identified.

**Conclusions:**

Malakoplakia is uncommon but the diagnosis should be considered where soft tissue masses develop at the site of chronic or recurrent infections. Obtaining tissue for histological examination is key to making the diagnosis. This case suggests that surgical resection is not always needed to achieve a good clinical and radiological outcome.

**Supplementary Information:**

The online version contains supplementary material available at 10.1186/s13256-023-03883-4.

## Background

Malakoplakia (or von Hansemann’s disease) is a rare condition characterized by inflammatory soft tissue masses, often arising at the site of recurrent or chronic infections. The condition and its characteristic histological features were first described in 1902 [1, 2]. In the following 60 years, there were fewer than a hundred cases reported, mostly malakoplakia of the bladder [3, 4]. The condition was subsequently described outwith the urinary tract [5, 6].

Insights into pathogenesis come from the detailed investigation of a young man with recurrent infections and subsequently disseminated *E coli*, in whom malakoplakia was demonstrated in both rectal tissue and a retroperitoneal mass [7]. He was found to have abnormal monocytes displaying impaired bactericidal activity against *E. coli*. These cells had “abnormal large lysosomal granules; low levels of cyclic-GMP in the mononuclear cells; and poor release of β-glucuronidase from leukocytes upon their exposure to opsonized zymosan particles” [7]. These defects corrected when the patient was given the cholinergic agonist bethanechol chloride. The patient’s recurrent infections stopped, his fistulae closed, and he gained weight. Similar defects in neutrophils in Chediak–Higashi Syndrome can be corrected using cholinergic agonists, cyclic guanosine monophosphate (GMP), or ascorbate.

There have been no randomized controlled trials, so case reports and our limited understanding of pathophysiology guide clinical practice. The case presented below, which has a number of unique features, is our contribution to this body of knowledge.

## Case presentation

The patient, a right-handed British Indian woman in her late 30s, presented with a week’s history of left foot weakness. She also had marked pain in the left leg, felt predominantly in the calf, and an area of numbness over the posterior aspect of her left thigh. These symptoms had worsened over the preceding 3 weeks. At onset of calf pain, she had attended an emergency department where she was found to have an elevated D-dimer (1172 ng/ml). Peripheral deep vein thrombosis was excluded using ultrasound.

Other symptoms included 1 month of bilateral ankle pain and 3 months of lethargy and poor appetite. She had lost 6 kg in weight over this period. She was constipated but had no urinary symptoms. There was no loss of perineal sensation. She reported occasional sweats but no true night sweats. There was no history of cough.

### Past medical history

Four years earlier, the patient had a caesarean section, complicated by retained products of conception, that is, placental and or fetal tissue that remained in the uterus after delivery, with consequent intrapelvic sepsis and abscess formation. She had required admission to intensive care and received, in total, 3 months of empiric, broad-spectrum antibiotics. No organism was isolated. Past medical history also included hypothyroidism and a congenital squint. The patient had a birthmark on her left buttock and, in her early 20s, had noted atrophy of the underlying adipose tissue. There was no history of recurrent infections. Admission medications were levothyroxine, prednisolone 40 mg daily (which we discontinued), omeprazole, plus recent use of codeine and amitriptyline for pain. She reported no drug allergies.

### Social and family history

The patient was born near London, was of Indian descent, and lived with extended family. Her contacts were well. She worked in an office and had previously been fully independent. She did not smoke, drank occasional alcohol, and had never used recreational drugs. She had traveled to India three times—3 months living with relatives as a young child, then two short holidays, the most recent being a decade earlier. There was no relevant family history.

### Examination

On examination, she appeared to be in considerable pain. She was afebrile with a blood pressure of 125/86 mmHg, a regular pulse at 81 beats per minute, a respiratory rate of 20 breaths per minute, and oxygen saturation of 100% in room air. Respiratory, cardiovascular, and abdominal examinations were unremarkable. There was no palpable lymphadenopathy. Other than her known squint, cranial nerves and upper limbs examined normally. Tone in her lower limbs was normal. The patient was unable to plantar or dorsiflex, invert or evert her left ankle (power 0/5). Left knee flexion was also weak (4/5) but power in her lower limbs was otherwise intact. The left ankle jerk was absent, otherwise all deep tendon reflexes were normal. Plantar reflexes were downgoing bilaterally. There was an area of hyperpathia over the left foot and decreased light-touch sensation over the posterior aspect of the left thigh. Vibration sense and proprioception were preserved. Firm pea-sized lumps were palpable underlying the skin covering the left buttock and sacrum. Examination findings were consistent with a complex foot drop, such as a lumbosacral radiculopathy or plexopathy.

### Baseline investigations

Results of baseline investigations are presented in Table 1. The admission chest x-ray was unremarkable. Urgent magnetic resonance imaging (MRI) of the lumbar spine and pelvis was organized and the patient admitted for investigations.

**Table 1:** Results of baseline investigations, with normal ranges in parentheses

Hemoglobin	106 g/L (115–165 g/L)	Immunoglobulins	Polyclonally raised IgA
Mean cell volume	88.3 fL (78–97 fL)		and IgG
Platelet count	380 × 10^9^/L (150–450 × 10^9^/L)	ANA	Negative
White cell count	3.8 × 10^9^/L (4–11 × 10^9^/L)	ANCA	Mixed weak pANCA
Neutrophil count	2.9 × 10^9^/L (1.5–4.0 × 10^9^/L)		and cANCA pattern
Lymphocyte count	0.6 × 10^9^/L (1.1–4.0 × 10^9^/L)	Anti-Myeloperoxidase	Negative
Sodium	137 mmol/L (133–146 mmol/L)	Anti-proteinase III	Negative
Potassium	4.5 mmol/L (3.5–5.3 mmol/L)	Rheumatoid factor	< 20 IU/mL (0–20 IU/mL)
Urea	6.0 mmol/L (2.5–7.8 mmol/L)	Anti-CCP	4.0 IU/mL (0–7 IU/mL)
Creatinine	57 µmol/L (44–80 µmol/L)	Alpha 1 antitrypsin	Not deficient
Bilirubin	7 µmol/L (0–21 µmol/L)	Ca 125	10 kIU/L (0–35 kIU/L)
Alanine aminotransferase	24 IU/L (0–40 IU/L)	hCG	< 1.0 IU/L 50–174 IU/L
Alkaline phosphatase	88 IU/L (30–130 IU/L)	Serum ACE	59 IU/L (16–85 IU/L)
Albumin	35 g/L (35–50 g/L)	Blood cultures^a^	No growth
Calcium (adjusted)	2.44 mmol/L (2.20–2.60 mmol/L)	Interferon Gamma Release Assay (Quantiferon)	Nonreactive
Phosphate	0.74 mmol/L (0.80–1.50 mmol/L	IgG4	< 0.42 mmol/L (0–1.3 mmol/L)
C-reactive protein	12 mg/L (0–5 mg/L)	HIV serology	Negative

^a^BD BACTEC system (Becton, Dickinson and Company, Franklin Lakes, New Jersey, USA), incubated in both aerobic and anaerobic conditions for 5 days

*ANA* Antinuclear Antibody, *ANCA* Anti-Neutrophil Cytoplasm Antibodies, *anti CCP* Anti–Cyclic Citrullinated Peptide, *Ca 125* Cancer antigen 125, *hCG* Human Chorionic Gonadotropin, *ACE* Angiotensin Converting Enzyme, *IgG4* Immunoglobulin G4, *HIV* Human Immunodeficiency Virus

### Cross-sectional imaging

Over a 2-month period following her caesarean section, four contrast-enhanced computed tomography (CT) scans of the abdomen and pelvis were performed at a local District General Hospital. Imaging demonstrated findings consistent with a postpartum uterus alongside a large enhancing intraabdominal and pelvic collection containing multiple locules of gas.

At this time, the pelvic soft tissue appeared slightly asymmetrical with loss of fat planes within the left hemipelvis and presacral regions and ipsilateral iliac chain lymphadenopathy. Chronic changes including soft tissue thickening, stranding, and calcification within the subcutaneous tissues of the right buttock were noted.

**Fig. 1:**
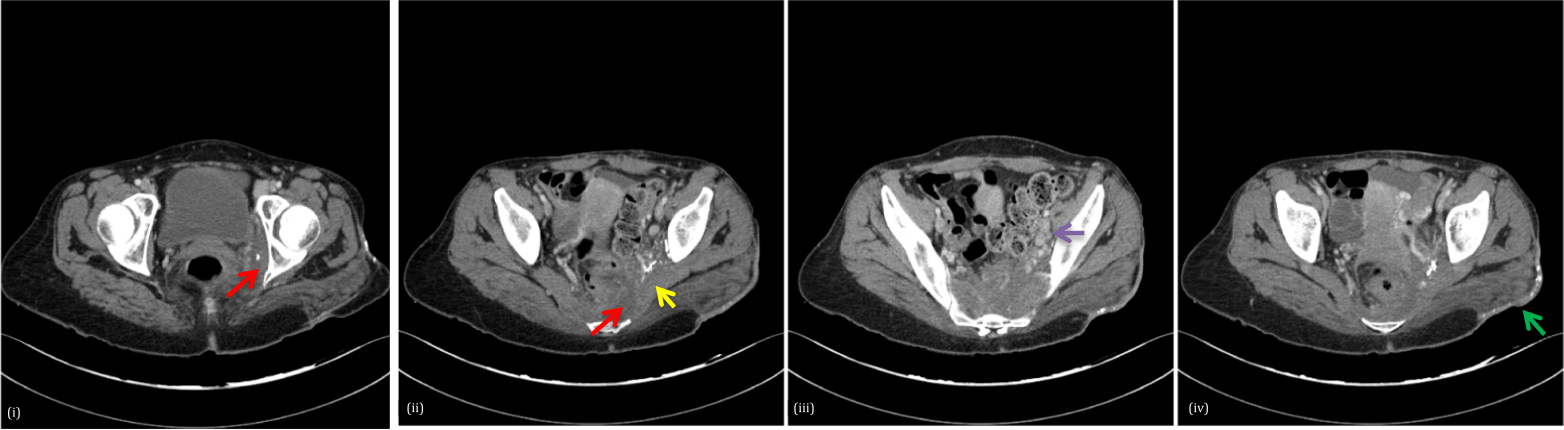
Computed tomography of the thorax, abdomen, and pelvis with contrast performed when the patient presented with foot drop. Serial axial computed tomography images: **(i)** and **(ii)** demonstrate an infiltrative heterogeneous soft tissue mass-like abnormality involving the left pelvic sidewall and presacral space (red arrows) intimately involving the left obturator internus and piriformis muscle (yellow arrow). Dystrophic areas of calcification are seen within the soft tissue mass. Despite the ill-defined and infiltrative nature, there is no associated bony destruction. Image **(iii)** depicts left iliac chain lymphadenopathy (purple arrow); image **(iv)** shows a loss of the normal subcutaneous fat, soft tissue thickening and multiple, punctate calcifications overlying the left gluteal maximus muscle, suggesting a chronic process (green arrow)

**Fig. 2:**
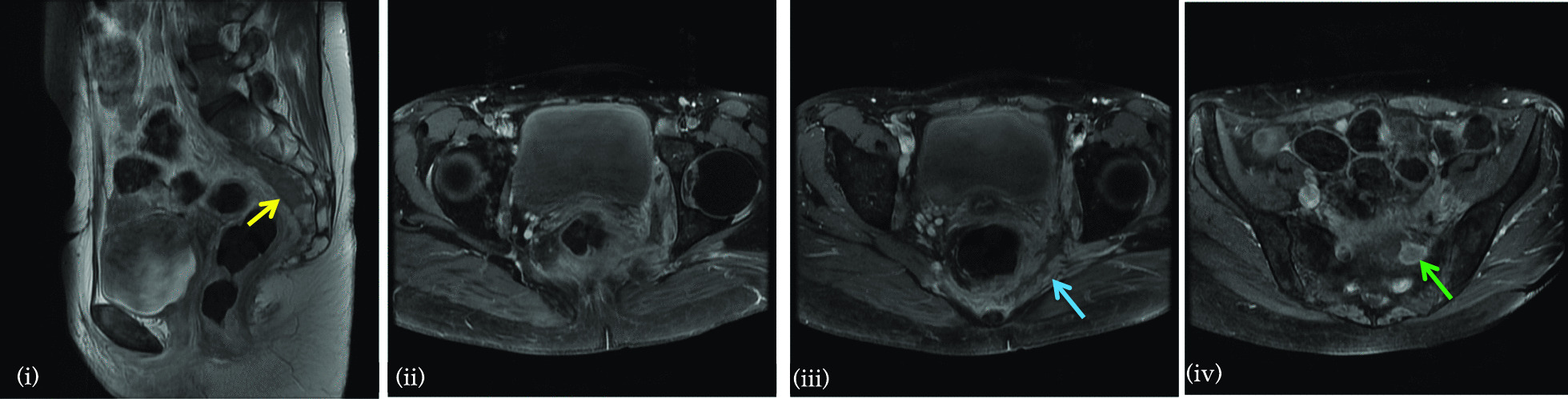
Magnetic resonance imaging of the pelvis with contrast performed for further evaluation of the soft tissue abnormality seen on computed tomography (Fig. 1). **(i)** Sagittal T2WI demonstrates soft tissue thickening in the presacral space (yellow arrow). Post-contrast fat-saturated axial T1WI (**(ii)**) and **(iii)** demonstrate enhancing soft tissue in the left pelvic side wall, left mesorectal fat, and extending through the greater sciatic foramen (blue arrow). **(iv)** Soft tissue could be seen to extend toward the left L5/S1 exiting neural foramen with involvement and thickening of the exiting left-sided sacral nerve roots (green arrow), lumbosacral plexus and ipsilateral sciatic nerve

The findings present on the CT (Fig. 1) and MRI studies (Fig. 2) during the presentation with foot drop can retrospectively be appreciated on the earlier CT studies. It is difficult to assess the extent of presacral soft tissue on these earlier CT scans, thus difficult to infer whether there was sacral foramina or sacral nerve root infiltration at that time. Combining the clinical presentation and radiological findings, it seems possible that a presacral soft tissue mass was present 4 years earlier. However, the presence of soft tissue within the hemipelvis/presacrum with associated lymphadenopathy could also be part of, or a reaction to, the concurrent collection.

### Subsequent management

Imaging was reviewed in the local gyne-oncology multidisciplinary team (MDT) meeting and by the regional sarcoma MDT. Appearances were felt not to be typical of either gynecological malignancy or sarcoma. The patient’s neuropathic pain proved difficult to manage.

There were multiple attempts to obtain tissue. Examination of tissue obtained through a radiologically guided biopsy of the presacral mass showed fat necrosis and features of chronic inflammation. Nothing grew from prolonged bacterial and mycobacterial tissue cultures. Bacterial cultures were incubated in both aerobic and anaerobic conditions for 10 days. Mycobacterial cultures were incubated in BD BACTEC Myco/F Lytic bottles for 42 days (Becton, Dickinson and Company, Franklin Lakes, New Jersey, USA).

A laparoscopic attempt to biopsy the presacral mass had to be abandoned as extensive adhesions meant the risks of proceeding were too great. A biopsy of the skin lesion on the left buttock showed fat necrosis and features of chronic inflammation, with a single calcified nodule also observed. Again, there was no growth from prolonged bacterial and mycobacterial tissue cultures.

Four weeks after admission, we had no diagnosis and, in view of persistent troublesome symptoms, empiric therapy for tuberculosis (TB) was initiated. Shortly afterward, a decision was taken to proceed to open biopsy of the presacral soft tissue mass.

### Histology

**Fig. 3:**
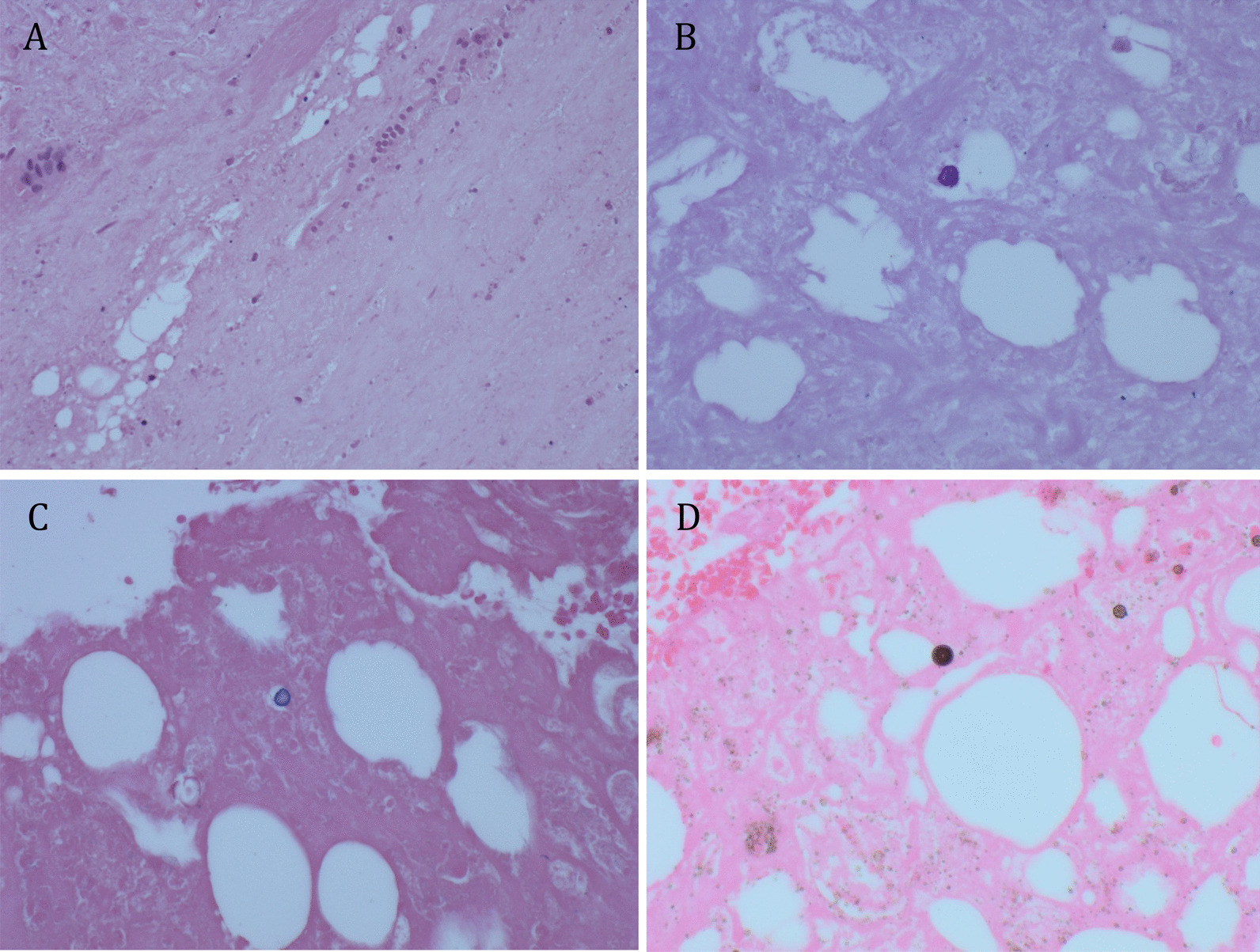
Histological examination of the soft tissue mass including **a** hematoxylin and eosin staining demonstrating fat necrosis plus the presence of histiocytes and giant cells, **b** periodic acid-Schiff staining demonstrating well demarcated “globules,” **c** Perls Prussian Blue staining, demonstrating that these globules contain iron, and **d** von Kossa stain, consistent with the globules containing calcium

Key histological findings are shown in Fig. 3. As in the other samples, fat necrosis and features of chronic inflammation were apparent. Histiocytes and giant cells were seen but there were no true granulomas. Well-demarcated globules were noted on Periodic acid-Schiff staining. Staining with Perls Prussian Blue and von Kossa stain demonstrated that these globules contained iron and calcium. Macrophages containing large intracytoplasmic inclusion bodies with calcium- and iron-laden lysosomal material have been termed Michaelis–Gutmann bodies and are the histological hallmark of malakoplakia [1].

### Treatment

Once the histology was reported, the patient was started on oral ciprofloxacin 500 mg twice daily, oral bethanechol 10 mg three times daily, and oral ascorbic acid 500 mg three times daily. Treatment for tuberculosis was continued, though this diagnosis was now felt to be less likely.

### Clinical and radiological response

**Fig. 4:**
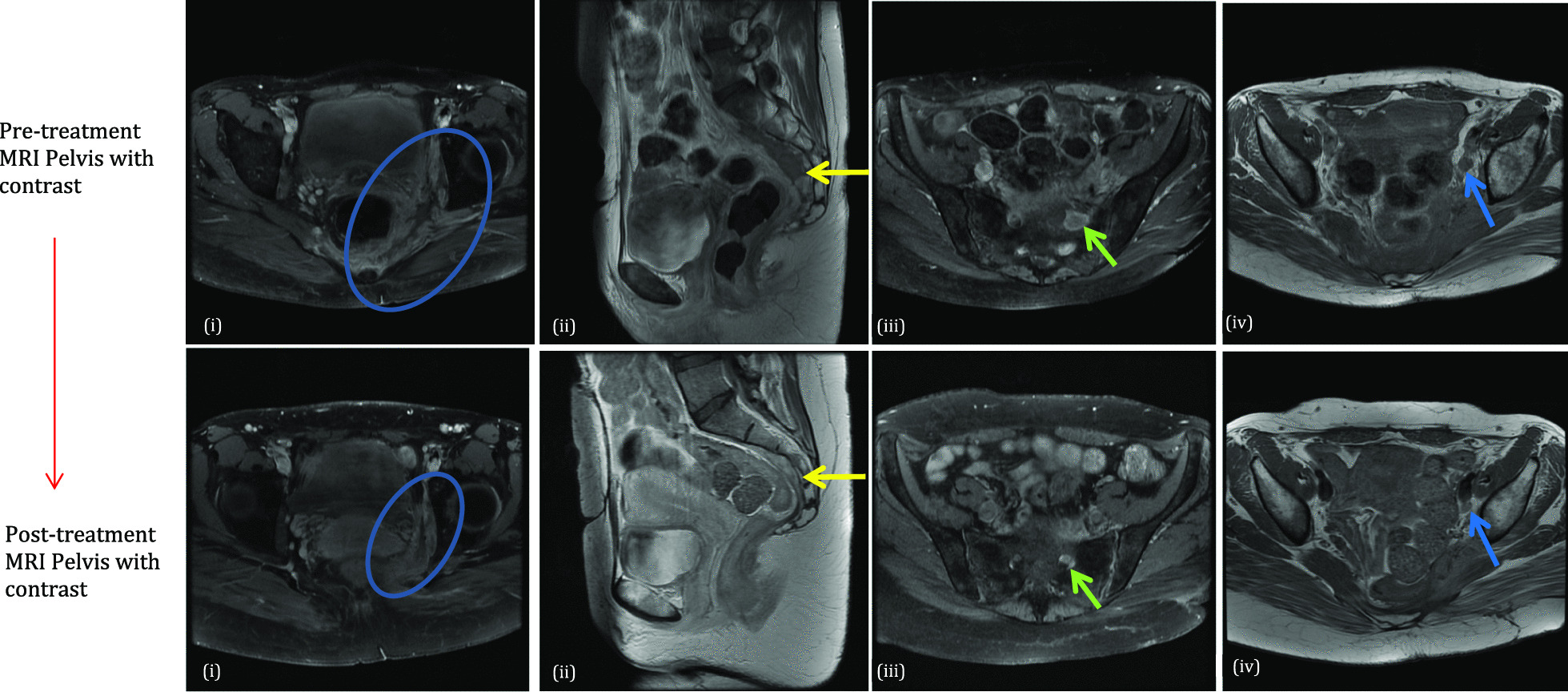
Magnetic resonance imaging scans of the pelvis obtained before and after her initial course of treatment. **(i)** Axial post-contrast fat-saturated T1WI and **(ii)** Sagittal T2WI show a significant reduction in the volume of soft tissue within the left hemipelvis (blue circles) and presacral space (yellow arrows). **(iii)** Axial post-contrast fat-saturated T1WI shows residual but less extensive enhancing soft tissue along the sacral nerve roots (green arrows). This is seen to continue to involve the exiting nerve roots, lumbosacral plexus and sciatic nerve—the thickness of these are, however, reduced. **(iv)** Axial T2WI shows reduced-volume left iliac chain lymphadenopathy (blue arrows)

The patient’s constitutional symptoms resolved quickly. She returned to her baseline weight within several months. Her blood results normalized. Her weakness improved but did not fully resolve. She required pregabalin for ongoing neuropathic pain. An MRI pelvis performed 6 months after the pre-treatment study showed significant improvement (Fig. 4). She received a total of 6 months of TB treatment and 8 months of ciprofloxacin, bethanechol, and ascorbic acid with no reported side effects, then demonstrated a prolonged period of stability off therapy.

Two years after completing therapy she re-presented with a 2-month history of increasing pain in the left lower leg associated with night sweats, fatigue, and weight loss with anorexia. On examination, she had 5/5 power in all muscle groups except for unchanged 3/5 power on dorsi and plantar flexion at the left ankle. She had a new normocytic anemia (hemoglobin 85 g/L) and her C-reactive protein (CRP) had climbed from < 5 to 24 mg/L. Repeat imaging was performed. Fortunately, both contrast-enhanced CT and pelvic MRI showed no significant interval change with regards to the previously described left hemipelvic and presacral infiltrative soft tissue. There was a minor increase in the volume of left-sided iliac chain lymphadenopathy.

Clinical, biochemical, and radiological changes suggested a relapse of her malakoplakia. The same regimen of empiric ciprofloxacin, bethanechol, and ascorbic acid was reinitiated.

She again had good resolution of her constitutional symptoms and improvement in her leg pain. Her anemia and CRP normalized. Follow-up pelvic MRIs at intervals of 4 and 7 months showed progressive nodal size reduction and ongoing stability of the pelvic soft tissue. Therapy was stopped after 6 months. She will be monitored as an outpatient.

### Genetic investigations

Whole exome sequencing (Novogene, Europe) was undertaken in the patient and her parents. Raw read data at > 30× coverage was processed and aligned to the Human Reference Genome version 19 (hg19) assembly according to best practices (https://github.com/sgul-genetics-centre-bioinformatics/Next-Generation-Sequencing-Pipelines). Candidate exonic variants were sought and prioritized according to low population frequency (≤ 0.01) and strong evidence for predicted functional consequences (Combined Annotation Dependent Depletion score ≥ 20) following *de novo* or recessive inheritance patterns (Additional file 1: Table S1). No standout causal variant was identified.

## Discussion and conclusion

We have described a case of presacral malakoplakia in an apparently immunocompetent young woman. Fewer than 100 cases of malakoplakia outwith the renal tract have been described in the medical literature [6]. To our knowledge, this is the first patient with malakoplakia in whom whole exome sequencing has been undertaken.

The literature describing malakoplakia of the urinary tract and at other sites has been reviewed elsewhere [5, 6]. The disease should be considered in patients with undiagnosed soft tissue masses. Diagnosis requires obtaining tissue for histological examination [5]. Treatments used in cases of malakoplakia include surgical resection, antibiotics, bethanechol chloride, and ascorbic acid [6].

Associations between malakoplakia and specific pathogens have been suggested: most commonly* Escherichia coli* and other gram-negative bacteria [6], but also *Mycobacterium tuberculosis* [8–10], *Rhodococcus equi* in immunocompromised patients [11], *Herpes simplex* in malakoplakia of the central nervous system [12], and several other pathogens [6]. An association between malakoplakia of the colon and colorectal adenocarcinoma has also been suggested [13–16]. Importantly, malignancy and malakoplakia can coexist in the same specimen [13–17]. Pathologists need to be alert to this possibility or important diseases may go undiagnosed. Cancer may be incorrectly staged if radiologists are unaware that part of a mass, or even regional lymphadenopathy [15], may be malakoplakia rather than metastatic spread.

As outlined below, many features of our case are consistent with other published case reports. However, several features of this case are noteworthy.

First, as in many other reported cases, there was a history of a significant infectious or inflammatory process at the site of disease. While no organism was isolated during our patient’s episode of intrapelvic sepsis, it seems likely that enteric flora were predominant.

Second, the possibility that the soft tissue mass was apparent on imaging from 4 years earlier suggests that the disease process may have been indolent.

Third, the subcutaneous tissue underlying the patient’s birthmark had similar histological and radiological features to the presacral mass. Fat loss at this site was reported to have predated the episode of intrapelvic sepsis, raising the possibility that any immune defect was either present from birth or acquired prior to her caesarean delivery. Cutaneous malakoplakia has been previously reported [18, 19]. We were not able to identify a genetic cause in this patient.

Fourth, while malakoplakia of the central nervous system is described [12], we were unable to find reports of malakoplakia causing radiculopathy, plexopathy, or involving other parts of the peripheral nervous system. As described in Fig. 2, we believe the pathology in this case to be the result of extrinsic compression rather than a problem arising from a peripheral nerve or associated structure.

Finally, pelvic adhesions made achieving a histological diagnosis challenging and meant we did not pursue surgical resection. While some advocate resection where feasible [6], our patient’s good response to medical management suggests surgery is not always necessary. Successful medical management of malakoplakia has also been reported by others [20–25]. However, our patient’s apparent relapse and subsequent good response to a second course of therapy suggests cure was not initially achieved. Whether further courses of treatment, or surgery, will be needed remains unclear.

Our understanding of malakoplakia remains limited. Unanswered questions include the underlying cause of the immune defect and whether it is inherited or acquired, the true prevalence of the condition given many cases must remain undiagnosed, the role for surgery, and the optimal duration of antibiotics and other medical therapies.

## Supplementary Information

**Additional file 1: Table S1.** Candidate exonic variants in our patient, with low population frequency (≤ 0.01) and strong evidence for predicted functional consequences (CADD ≥ 20) following de novo or recessive inheritance patterns. Population frequencies ascertained in the following population cohorts: 1000 Genomes, Exome Sequencing Project, gnomAD, ExAC and the Greater Middle East variome project.

## Data Availability

We do not have consent to share any more data about this patient than is included in the manuscript. A link to the sequencing pipelines that were used is provided in the text. No other code was used in preparing this manuscript.

## Abbreviations

CRP: C-reactive protein
CT: Computed tomography
Cyclic GMP: Cyclic guanosine monophosphate
hg19: Human Reference Genome version 19
MDT: Multidisciplinary team
MRI: Magnetic resonance imaging
TB: Tuberculosis
