# Supplementary material for: Presacral malakoplakia presenting as foot drop: a case report
Source: J Med Case Rep. 2023 Apr 7;17:154. doi: 10.1186/s13256-023-03883-4 (PMC10080903; doi:10.1186/s13256-023-03883-4)
Supplement: Supplementary file 1 — Additional file 1: Table S1. Candidate exonic variants in our patient, with low population frequency (≤ 0.01) and strong evidence for predicted functional consequences (CADD ≥ 20) following de novo or recessive inheritance patterns. Population frequencies ascertained in the following population cohorts: 1000 Genomes, Exome Sequencing Project, gnomAD, ExAC and the Greater Middle East variome project. [file 13256_2023_3883_MOESM1_ESM.docx]

| **Chr** | **Start** | **End** | **Ref** | **Alt** | **Gene.refGene** | **Change** |
| --- | --- | --- | --- | --- | --- | --- |
| 1 | 7889973 | 7890026 | AGAATCCATCCCATCCTACTGCCAGCGCTCTGTCCACAGGATCGCCTCCCATGA | - | PER3 | p.E988Gfs*192 |
| 1 | 26608866 | 26608867 | CC | - | UBXN11 | p.G376Sfs*31 |
| 1 | 26608869 | 26608896 | GGACTGGGGCCGGGACCGGGACCGGGAC | - | UBXN11 | p.C366Sfs*32 |
| 3 | 27763427 | 27763427 | G | C | EOMES | p.A120G |
| 3 | 100170600 | 100170600 | - | TCCTAGAAGGCATTCTCATGAGGACCAGGAATTCCGATGCCGATCGTCTGACCGTCT | LNP1 | p.S80_H81ins |
| 4 | 86950379 | 86950379 | C | A | MAPK10 | p.G408V |
| 4 | 178357439 | 178357439 | T | C | AGA | p.K230R |
| 6 | 30521156 | 30521156 | T | C | GNL1 | p.D260G |
| 6 | 31106501 | 31106501 | - | C | PSORS1C1 | p.H40Pfs*3 |
| 6 | 31630204 | 31630204 | G | A | GPANK1 | p.P304S |
| 6 | 31632246 | 31632246 | G | A | GPANK1 | p.P4S |
| 6 | 32037938 | 32037938 | C | A | TNXB | p.K1748N |
| 6 | 32632749 | 32632749 | A | C | HLA-DQB1 | p.Y69D |
| 7 | 47342790 | 47342790 | G | A | TNS3 | p.T1072M |
| 7 | 127014333 | 127014333 | C | G | ZNF800 | p.E353Q |
| 13 | 30077257 | 30077257 | A | G | MTUS2 | p.I321V,MTUS2 |
| 16 | 426769 | 426769 | G | A | TMEM8A | p.R227W |
| 16 | 88494603 | 88494603 | G | T | ZNF469 | p.S242I |
| 19 | 35250222 | 35250222 | G | A | ZNF599 | p.T495I |
| 19 | 55785915 | 55785915 | C | T | HSPBP1 | p.R210Q |
| 19 | 58788583 | 58788630 | GGCCAGAAGGATCTGAGAGAGTGTGGCCAGCTTGAGCCCTCAGGAGTC | - | ZNF544 | p.R93R |
| X | 31514999 | 31515001 | AGA | - | DMD | p.L89Vfs*868 |
| 14 | 23612372 | 23612372 | T | G | SLC7A8 | p.T79P |

**Table S1 - Candidate exonic variants in our patient, with low population frequency (≤0.01) and strong evidence for predicted functional consequences (CADD≥20) following de novo or recessive inheritance patterns. Population frequencies ascertained in the following population cohorts: 1000 Genomes, Exome Sequencing Project, gnomAD, ExAC and the Greater Middle East variome project**
